# The Waves Defied

**Published:** 1875-10

**Authors:** 


### THE WAVES DEFIED.

If we could have had our way, we should have caused the introduction of one feature to the Institute Fair which would have added largely to the attractiveness of that splendid exhibition, and would have extended the sphere of usefulness of one of the most vauable appliances which we have ever seen. We *should* have had our way if the grand idea had occurred to us in time. If we had thought of it during any of the early weeks of preparation in the great building where the Fair is held, the thing could have been done. We refer to the exhibition daily, or at stated intervals during the Fair, of Capt. Stoner's Life Suit. To give a good exhibition of it, the tank in the centre of the hall would require to be sunk to a depth of say five or six feet. This would enable a child to put on one of the impervious suits and float about; and even this imperfect test of the security and saving power of the appliance would be productive of good results.

This life-dress is one of the most valuable inventions—perhaps *the* most valuable—to be found anywhere. It is an absolute preserver of life in the event of wreck or fire or any form of sea-accident, by which the vessel is destroyed. Had the Schiller, or any of the lost fleets of past years, been amply provided with these suits, not one life—except, perhaps, some infants'—need have been lost. Scarcely one of all the ships which in the past have sailed silently away and have never afterwards furled their wings and come to the shore again, would have gone down unstoried, with no survivor left to tell the dismal tale of collision, of ice-pack, of breakers, of hidden rock, of fire, or of destructive gale, had each and all borne these suits as a part of the outfit. With the history of the past strewn with wrecks of preciously freighted ships, who shall say that any device which robs shipwreck of its chief terrors, is not one of vast importance and priceless value?

This suit, which so defies the waves, is simple enough. It depends for its excellence upon that wonderful gum, India-rubber. It is only a suit of clothes in one garment, made from cloth which has been coated with this elastic and impervious gum; and then so vulcanized that whether hot or cold, its flexibility is maintained. Attached to the suit, at the waist, are twelve air-chambers, any two of which are ample to sustain a human being in the water. Straps pass over the shoulders to the feet, and to these straps weights are fastened. These keep the feet down, and thus the attitude of the body in the water is precisely that of a person standing. The man or woman becomes a perfect ship, floated by the buoyant power of the air-cells, and ballasted by the weights. The suit covers all ordinary clothing except boots and hat. The head is protected, all but the face, by a close-fitting hood. A pillow is provided in the form of a hollow cape, which is inflated at will. In a quiet sea there would be no difficulty in sleeping. The body never gets chilled while in this suit, because the clothes are dry, and the heat from the body is all confined. This is the suit; but lest the shipwrecked voyager should remain unrescued for several days, he is supplied with provisions which also float in a tight compartment near at hand. To signalize passing ships, this float has also stowed away, sky-rockets and materials for igniting them, and signal lights, together with a flag-staff and flag.

We have seen this suit worn in a heavy sea, and have felt that we would never take a long voyage on the ocean without having one at hand. If we owned Congress, we would compel it to pass a law making it a crime for a vessel to go to sea without a full supply of these suits. The owners of this affair are the American Life-Saving Suit Company, having an office at No. 52 Broadway. They receive applications from all who are about to depart for foreign shores, for the use of these suits. For the sum of $5, a suit is placed in the state-room of the passenger, and the steward in charge gives the simple instructions which would be useful to those who had never seen the suit worn. To our minds, a sea-voyage would be shorn of its chief terrors, with the knowledge that one of these suits was in our state-room. It seems as if nearly all passengers must feel pretty much as we do in this matter. If they do, they will not venture far from land without this best of life-preservers.

